# Pain perception and functional/occlusal parameters in sleep bruxism subjects following a therapeutic intervention

**DOI:** 10.1186/s13005-019-0188-6

**Published:** 2019-01-29

**Authors:** Michelle Alicia Ommerborn, Rita Antonia Depprich, Christine Schneider, Maria Giraki, Matthias Franz, Wolfgang Hans-Michael Raab, Ralf Schäfer

**Affiliations:** 1 Department of Operative Dentistry, Periodontics, and Endodontics, Faculty of Medicine, Heinrich-Heine-University, Moorenstr. 5, 40225 Düsseldorf, Germany; 2 Department of Cranio- and Maxillofacial Surgery, Faculty of Medicine, Heinrich-Heine-University, Düsseldorf, Germany; 3 Clinical Institute of Psychosomatic Medicine and Psychotherapy, Faculty of Medicine, Heinrich-Heine-University, Düsseldorf, Germany

**Keywords:** Sleep bruxism, Pain perception, Randomized controlled trial, Occlusal splint, Cognitive therapy, Craniomandibular function

## Abstract

**Background:**

This study was conducted to assess the individual pain perception in sleep bruxism (SB) subjects. Moreover, the effects of a cognitive behavioural therapy (CBT) compared to an occlusal appliance (OA) on pain perception and a possible continuative impact on several functional parameters were investigated.

**Methods:**

A total of 57 SB subjects participated in this investigation. The diagnosis of SB was based on the clinical criteria of the American Academy of Sleep Medicine (AASM). Twenty-eight SB subjects were randomly allocated to the CBT group and 29 to the OA group. The therapeutic intervention took place over a period of 12 weeks, whereby both groups were examined at baseline, immediately after termination of the intervention, and at a 6-month follow-up for pain perception and functional parameters. At each of the three measurement periods, participants completed the pain perception scale and ten functional/occlusal parameters were recorded.

**Results:**

Of the 12 parameters recorded, statistically significant main effects were found for the affective pain perception (*p* < 0.05) and for the three functional variables. Interestingly, the values obtained for the affective pain perception were considerably below that of a reference group. Apart from the determined statistically significant results, the values recorded for all functional/occlusal variables as well as those obtained for the sensory pain perception were clearly located within normative ranges.

**Conclusions:**

Within the limitations of this study, it might be concluded that the significantly reduced affective pain perception in SB subjects is the expression of an adaptation mechanism.

## Background

According to a recently published international consensus on the assessment of bruxism, sleep and awake bruxism have been defined as “masticatory muscle activities that occur during sleep (characterised as rhythmic or non-rhythmic) and wakefulness (characterised by repetitive or sustained tooth contact and/or by bracing or thrusting of the mandible), respectively” [1]. Although a voluminous awareness on the topic of sleep bruxism (SB) has been generated during the past three decades, a detailed clarification regarding the aetiology of SB and, consequently, the development of an effective treatment approach are still lacking. Current efforts/approaches to explain the aetiology of SB focus on an altered expression of D2-receptor binding [2], transient sleep arousals [3, 4], predisposing personality traits or stress [5–7]. However, in terms of a multifactorial genesis, a combination or the interaction of several central factors appears to be responsible for the onset or the modulation of SB activity [8].

Considering the relationship between masticatory muscle activities, such as bruxism, and temporomandibular disorders (TMDs), several studies indicate that the former are risk factors for the development of specific subgroups of TMDs [9–11]. However, a distinct clarification of the necessary conditions or predisposing factors that initiate the shift from a masticatory muscle activity into a manifest TMD is a matter of current scientific efforts. According to the stress-muscular hyperactivity model, some authors found parafunctional behaviours, especially those that increase muscle tension, and emotional states to be good predictors of jaw pain levels in patients with TMD and healthy controls [12]. A contrary reasoning has been provided by means of a study showing a negative association between tooth-grinding and palpated pain severity. The authors concluded that the obtained negative association between tooth grinding and pain severity, as predicted by an adaptation model of face pain, would cast serious doubt on the theory that myofascial face pain is maintained by tooth-grinding [13]. Moreover, personality characteristics as differential variables of pain perception have been analysed with respect to the use of passive or active coping strategies. The authors were able to show that subjects with chronic pain and high scores in neuroticism use passive coping strategies whose inefficiency is reflected in a greater intensity of perceived pain [14]. Furthermore, results of other scientific investigations revealed that changes in mood affect the pain perception [15] or may influence the magnitude of pain threshold increase [16].

Further components which are known to have an adverse impact on the intensity of perceived pain in TMD-patients are depressive symptoms [17] as well as the application of maladaptive coping strategies [18–20]. Moreover, the outcome of several investigations refers to an association of both depressive symptoms [10, 21] and maladaptive coping styles with SB [22]. Keeping in mind the aforementioned, it appears surprising that to date little is known about the pain perception in SB subjects. This in particular is astonishing, as the individual pain perception with its sensory and affective components was found to be an important element of the comprehensive assessment of the multidimensional pain process [23]. In this respect, a randomized controlled trial (RCT) evaluating the effect of a cognitive behavioural therapy (CBT) vs. a standard occlusal appliance (OA) therapy on the individual pain perception of SB subjects was conducted. Accordingly, the objectives of the present study were:

- Firstly, to generally assess the individual pain perception in SB subjects not presently being subjected to a treatment,
- Secondly, to investigate possible changes concerning the individual pain perception in sleep bruxers being subjected to either a CBT or to an OA therapy in a pre-treatment, post-treatment and a six-month follow-up design and,
- Additionally, to assess possible continuative effects of either of the applied management approaches, several functional parameters were also controlled.

## Methods

### Sample

The effects of an OA therapy compared with a CBT on SB activity were evaluated in a previously published randomized controlled clinical trial [24]. The materials and methods of the aforementioned study are briefly reported in the following section. Subsequent to a complex recruitment process, 57 SB subjects who fulfilled the predefined medical, psychological and dental inclusion criteria participated in the study. All subjects were recruited by means of announcements in local newspapers and placards on campus and were all German native speakers.

In order to verify the following inclusion and exclusion criteria, the screening process included a thorough dental examination and a semi-standardized psychological diagnostic interview [25]. One trained dentist performed all dental examinations and procedures and, accordingly, one experienced psychologist conducted the psychological diagnostic interview of each subject as well as the CBT. As applied in antecedent investigations [6, 7, 26–28], the diagnosis of SB was based on the clinical criteria of the American Academy of Sleep Medicine (AASM) [29, 30], which required the sleeping partner’s report of grinding sounds during the night in the last six months. Furthermore, the participants were called having at least one of the following symptoms: self-report of muscle fatigue or tenderness on awakening, the presence of tooth wear to at least the magnitude of dentine exposure [31] and masseter hypertrophy upon voluntary clenching [30, 32]. To obtain additional information whether SB subjects also reveal possible awake bruxism, participants were asked if they clench their teeth during the day [7, 33]. Subjects undergoing current dental or psychological treatment, having more than two missing molars (excluding third molars), or wearing a removable prosthesis or extensive fixed prosthetic restorations, were excluded from the present study. Moreover, to exclude serious psychological and psychiatric disorders, such as schizophrenia or manic or bipolar affective disorders, a semi-standardized psychological diagnostic interview of 90 min duration, according to the international diagnosis checklist of the International Classification of Diseases and Related Health Problems, 10th revision (ICD-10), was conducted for each subject. Further exclusion criteria were the use of psychotropic drugs, drug and/or alcohol abuse and the presence of central nervous system and/or peripheral nervous system disorders. Healthy adults, between 20 and 40 years of age, who fulfilled the criteria for SB diagnosis were included in this investigation and subsequently randomized to either the OA group (*n* = 29) or the CBT group (*n* = 28). Two employees of the Clinical institute of Psychosomatic Medicine and Psychotherapy randomly and consecutively assigned the subjects to each group by means of a draw. Both colleagues were not associated with this clinical investigation and were unaware of the subjects’ names or diagnoses.

All subjects provided written informed consent to the procedures approved by the Institutional Human Subjects Ethics Committee (Heinrich-Heine-University of Düsseldorf, Study No. 1497). Each subject received a financial compensation for his or her participation in this time-consuming investigation.

### Intervention

The treatment in both groups took place over a period of 12 weeks. At first, participants of both groups were counselled regarding SB, and in particularabout the respective treatment approach that was to beimplemented on them. Apart from this introduction, only the CBT group was subjected to a detailed counselling.

**Fig. 1:**
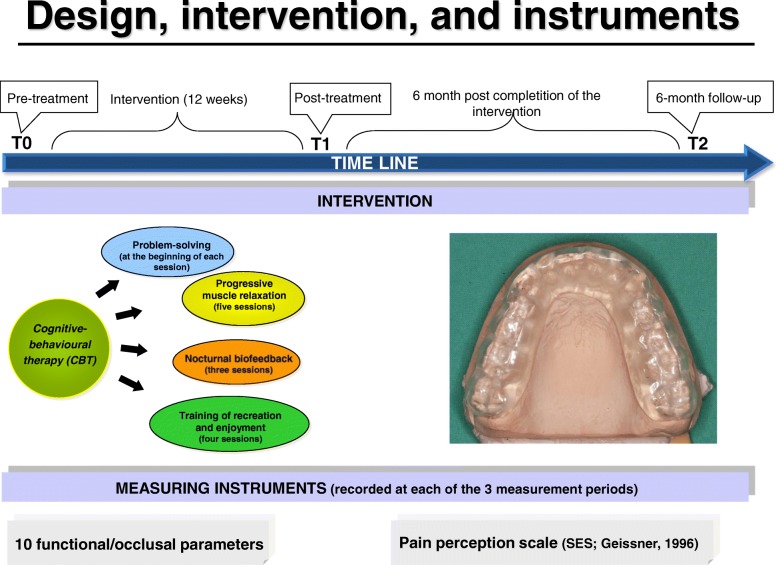
Schematic illustration of the study design and intervention composition

Details on the composition of the applied CBT have been reported in thorough in a previous article [24]. Briefly, the CBT was conducted weekly in groups comprisingan average of nine participants. A total of twelve meetings of 1,5 h duration were held. Following a comprehensive introduction to the topic of SB and the possible role of stress in its development, the participants of the CBT group received a modified stress-management training that was based on a preliminary published program [34]. This program was modified according to the specific requirements of SB subjects and it consisted of the modules problem solving, progressive muscle relaxation [35, 36], nocturnal biofeedback, training of recreation and enjoyment (Fig. 1).

As previously described in detail [24], the OA group received a hard acrylic stabilization splint, to be worn in the maxilla, with full coverage of the occlusal surfaces (Fig. 1). The OA provided even, simultaneous occlusal contacts of all mandibular supporting cusps, verified by a 12 μm thick articulation paper, and during laterotrusive and protrusive movements the canine guidance caused a disclusion of all teeth [37]. The participants were instructed to use the OS each night over a period of 12 weeks. After one and six weeks, the participants of the OA group were re-examined and if necessary the splint was adjusted again. All subjects were treated by one experienced dental clinician.

### Study design and dependent variables

After verification of the inclusion criteria and randomized allocation of the participants to either the CBT or the OA group, subjects were analysed at three measurement periods: the first examination took place immediately prior to initiation of the intervention (pre-treatment), the second examination was conducted subsequent to the termination of the 12 weeks intervention period (post-treatment), and in order to gain information with respect to a possible long-term effect the third one was carried out half a year after termination of the intervention period (6-months follow-up) (Fig. 1). Considering the last aspect, upon completion of the treatment period, the participants of the CBT group where instructed to further apply the stress-solving strategies and relaxation techniques which they had learned and trained within the 12-weeks therapy, but the participants of the OA group had to return their OA. To omit possible uncontrolled interactions or side effects resulting from other therapies, at the beginning of the recruitment it had been pointed out to each participant that additional SB therapies or extensive restorative procedures must be carried out after completion of the third measurement period. In addition, all participants were given the opportunity to try the other type of treatment after completion of the study, if desired.

To gain information about the individual pain perception and to compare it with that of an adequate reference group, subjects filled in the Pain Perception Scale (in German, Schmerzempfindungsskala, SES) [38]. It is a diagnostic instrument that allows multifaceted and standardized quantification of pain sensation. The questionnaire consists of 24 items (4 point response scale, 1–4) that ask about different characteristics of individual pain sensation. The items of the SES are attributed to five characteristics, two affective characteristics (general affectiveness and inveteracy) and three sensory characteristics (rhythmicity, local infiltration, temperature). The two affective characteristics of pain perception were combined to form the global dimension affective pain perception (14 items) and the three sensory characteristics were merged to give the global dimension sensory pain perception (10 items). The sensory characterization of pain includes the estimation of physical stimulus characteristics of pain perception (e. g. burning pain), whereas the affective characterization of pain combines the description of individuals suffering from pain (e. g. agonizing).

Regarding the appraisal of the recorded values, the user manual includes tables of a reference sample (*n* = 1048) or samples with specific chronic diseases. In addition to the raw data, these tables also list T-value equivalents (the mean of the T-distribution amounts to 50 and the standard deviation amounts to 10) of the two global dimensions and the three sensory characteristics, which are recommended to be used. For the interpretation of the two global dimensions, T-values between 40 and 60 were considered as the average pain characteristic, T-values between 30 and 39 as below average, between 61 and 70 as above average, and between 20 and 29 far below average.

Focussing on the assessment of pain qualities, the pain perception scale is nowadays considered the most established psychometric instrument in the German-speaking countries [39]. It is used internationally and is characterized by its good validity. Moreover, it has been proven suitable for group comparisons and the evaluation of therapeutic effects, as carried out in the present study. Thus, the global dimension affective pain perception revealed a Cronbach’s alpha of 0.92 and a two month re-test reliability of r = 0.96, the global dimension sensory pain perception a Cronbach’s alpha of 0.81 and a two month re-test reliability of r = 0.95. Report of the SES data includes a calculation of the two global dimensions and the three sensory characteristics.

In order to estimate possible somatic effects of either of the applied interventions, the following ten standard functional/occlusal parameters were recorded from each participant by means of a digital calliper: vertical (overbite) and horizontal (overjet) overlap of the maxillary and mandibular right central incisors, maximum active mouth opening, maximum active right and left lateral movement of the mandible, maximum protrusive movement of the mandible, the presence of a slide from centric occlusion (CO) to maximum intercuspation (MI) and, if present, the length of the slide from CO to MI. Details on the measurement of the slide from CO to MI have been published elsewhere [40], Moreover, the resiliency of the right and left temporomandibular joint (TMJ) was determined [41]. The measurement of these parameters was performed clinically and conducted by one trained dentist of the department.

### Statistical analysis

The statistical software SPSS version 19.0 (IBM Corp.) was used for the statistical analysis of the present data. In order to provide clear presented data, the levels of education were divided into three grades: x_1=_10 years school; x_2=_13 years school; x_3=_18 years school (university). Furthermore, anterior crowding in the mandible was classified on a five-point scale: 0 = no crowding; 1 = 1-3 mm of crowding; 2 = 3-5 mm; 3 = 5-7 mm; 4= > 7 mm.

Normal distribution was tested by using Shapiro-Wilk tests along with an assessment of histograms and normal QQ plots. The baseline characteristics from both study groups were compared for equality by means of independent samples *t*-test that was used for the analysis of the normally distributed quantitative variables. If data were not normally distributed, the non-parametric equivalent, the Mann-Whitney *U* test, was applied. When using the Mann-Whitney *U* test, the adequate statistical values are the mean ranks and the sum of ranks. However, to enhance the comparability of the reported functional parameters, data are given as means and standard errors of the mean (SEMs). Considering the nominal scaled variables, the presence of possible differences between the two treatment groups at baseline was verified by means of the Pearson χ^2^ test. Means and SEMs were calculated. Two-way analysis of variance (ANOVA) was conducted by using a general linear model (GLM) with repeated measures for the statistical analyses of a between subject factor (type of applied intervention, viz. OA or CBT) and within the three measurement periods as well as their interactions. Contrast analyses were performed to test differences between these three factor levels. For all repeated measures analyses, according to Greenhouse-Geisser ε-correction procedure for degrees of freedom were applied [42]. For all effect measures and contrast analyses partial eta^2^ (η^2^) effect size values were calculated which were the proportion of the total superpopulation variance (σ_t_^2^) made up by the variance of the population means (σ_m_^2^) [43]. If data were not normally distributed, nonparametric Friedman test together with Wilcoxon signed rank tests were applied and, accordingly, for dichotomous categorical variables nonparametric Cochrans Q-test was used. A significance level of *p* < 0.05 was predefined in all cases.

## Results

Analyses of the sociodemographic and further descriptive data of the 57 SB subjects included in the study revealed no statistically significant difference between the OA and the CBT group regarding age, gender, education, occlusal guidance, number of teeth, number of teeth with occlusal restorations, possible awake bruxism, and signs of lip or cheek biting (Table 1). Moreover, the Angle’s Classification of malocclusion, recorded on the right and the left side for the canines and for the first molars, [44] as well as the anterior crowding in the mandible which were identified from dental study casts [45, 46] showed no differences between the two groups. As a consequence of either missing canines or first molars in some patients the sample size varied between 57 and 43 participants. Furthermore, the recorded data of the SES and the functional/occlusal parameters except for the length of a slide from CO to MI also indicated no statistically significant baseline differences between the two treatment groups.

**Table 1:** Sociodemographic data and baseline characteristics of the included SB subjects treated either with OA or with CBT

	CBT group	OA group	*p*-value
Age (y) (*n* = 57)	28.50 (0.96)	29.41 (0.83)	0.47^a^
Female/Male ratio (*n* = 57)	19/9	20/9	0.93^b^
Education (*n* = 57)	1 × _1_; 20 × _2_; 7 × _3_	2 × _1_; 16 × _2_; 11 × _3_	0.44^b^
Canine guidance (%) (*n* = 57)	7.1	0	0.14^b^
Incisal guidance (%) (*n* = 57)	39.3	24.1	0.22^b^
Group guidance (%) (*n* = 57)	53.6	75.9	0.08^b^
Molar occlusion right side (*n* = 43)	5 Class I	8 Class I	0.22^b^
	6 Class II	13 Class II	
	7 Class III	4 Class III	
	10 Missing	4 Missing	
Molar occlusion left side (*n* = 45)	8 Class I	12 Class I	0.66^b^
	4 Class II	10 Class II	
	5 Class III	6 Class III	
	11 Missing	1 Missing	
Cuspid occlusion right side (*n* = 46)	8 Class I	8 Class I	0.54^b^
	9 Class II	18 Class II	
	1 Class III	2 Class III	
	10 Missing	1 Missing	
Cuspid occlusion left side (*n* = 46)	7 Class I	16 Class I	0.27^b^
	10 Class II	12 Class II	
	1 Class III	0 Class III	
	10 Missing	1 Missing	
Anterior crowding (*n* = 46)	0: *n* = 3	0: *n* = 6	0.29^b^
	1: *n* = 14	1: *n* = 19	
	2: *n* = 0	2: n = 3	
	3: n = 1	3: n = 0	
	10 Missing	1 Missing	
Number of teeth (*n* = 57)	28.25 (0.31)	28.48 (0.28)	0.78^c^
Number of teeth with occlusal restorations (*n* = 57)	9.89 (1,02)	10.45 (0,92)	0.69^a^
Possible awake bruxism (%) (*n* = 57)	45.9	54.9	0.51^b^
Signs of lip/cheek biting (*n* = 57)	5 yes/23 no	10 yes/19 no	0.15^b^

^a^Two-sample, two-tailed *t* test; data are presented as mean and standard error or the mean (SEM)

^b^Pearson χ^2^ test

^c^Mann-Whitney *U* test; data are presented as mean and SEM

### SES

Summon the whole, neither the three subtotals nor the two global dimensions of pain perception showed a significant difference between the OA and the CBT group as tested with the nonparametric Mann-Whitney *U* test.

The statistical analyses of the subtotal rhythmicity revealed a statistically significant effect ‘measurement period’ in the OA group (Friedman test, χ^2^ = 8.98, *p* = 0.011). A statistically significant decrease from pre-treatment to post-treatment (Wilcoxon signed rank test, *p* = 0.015) as well as a statistically significant increase from post-treatment to 6 months follow-up were observed (Wilcoxon signed rank test, *p* = 0.016). No statistically significant differences were measured between the two groups (Table 2).

**Table 2:** Descriptive statistics of the SES variables derived at the three measurement periods for both treatment groups

Variable	Treatment group	Measurement period		
		Pre-treatment	Post-treatment	6-months follow-up
Sensory characteristic rhythmicity^a^	CBT	4.59 (0.42)	4.33 (0.38)	4.59 (0.47)
	OA	4.38 (0.36)	3.52 (0.2)	4.28 (0.38)
Sensory characteristic local infiltration^a^	CBT	5.81 (0.59)	5.15 (0.25)	5.41 (0.34)
	OS	5.04 (0.37)	5.04 (0.34)	5.66 (0.43)
Sensory characteristic temperature^a^	CBT	3.48 (0.23)	3.15 (0.09)	3.37 (0.19)
	OA	3.35 (0.18)	3.21 (0.09)	3.66 (0.23)
Global dimension affective pain perception^a^	CBT	19.86 (1.35)	18.00 (1.16)	18.68 (0.82)
	OA	17.83 (0.97)	17.62 (1.13)	20.41 (1.5)
Global dimension affective pain perception^b^	CBT	38/ 16.1	37/ 9.8	38/ 12.1
	OA	37/ 9.8	37/ 9.8	38/ 16.1
Global dimension sensory pain perception^a^	CBT	13.89 (0.96)	12.63 (0.60)	13.37 (0.75)
	OA	12.76 (0.73)	11.76 (0.5)	13.59 (0.91)
Global dimension sensory pain perception^b^	CBT	43/ 33.2	42/ 27.5	42/ 27.5
	OA	42/ 27.5	40/ 18.9	43/ 33.2

^a^Friedman test together with Wilcoxon signed rank test; values are presented as mean and SEM of the raw data

^b^T-values and percentile ranks

The calculation of the global dimension affective pain perception showed a statistically significant effect ‘measurement period’ in the OA group (Friedman test, χ^2^ = 6.1, *p* = 0.047). Further analyses exhibited a statistically significant increase of the affective pain perception from post-treatment to 6 months follow-up (Wilcoxon signed rank test, *p* = 0.015). Considering the T-values, across all measurement periods and independent of the respective intervention, the obtained values ranged between 37 and 38. As T-values between 40 and 60 were categorised as average pain characteristic and T-values between 30 and 39 as below average, the T-values of the global dimension affective pain perception derived from the present study were located below average compared to the reference sample provided in the SES manual.

The analyses of the subtotals local infiltration and temperature and the global dimension sensory pain perception failed to display a statistically significant effect ‘measurement period’ (Table 2). T-values recorded for the global dimension sensory pain perception ranged at all measurement periods and independent of the respective intervention between 40 and 43 and were, thus, interpreted as average pain characteristic.

### Functional/occlusal parameters

Two-way ANOVA of the maximum active right lateral movement demonstrated a statistically significant main effect ‘measurement period’ (F (1.53/84.09) = 3.86, *p* = 0.035, η^2^ = 0.066). Contrast analyses revealed a significant increase of the maximum active right lateral movement from pre-treatment to post-treatment (F (1/55) = 7.16, *p* = 0.01, η^2^ = 0.115) in both groups (Table 3). No significant interaction between ‘measurement period’ and ‘group’ was observed.

**Table 3:** Descriptive statistics of the functional and occlusal parameters derived at the three measurement periods for both treatment groups

Variable	Treatment group	Measurement period		
		Pre-treatment	Post-treatment	6 months follow-up
Maximum active mouth opening (mm)^a^	CBT	50.55 (1.02)	50.89 (1.18)	50.7 (1,11)
	OA	49.91 (1.01)	50.4 (1.16)	49.52 (1.09)
Overbite (mm)	CBT (mm)	2.66 (0.23)	2.66 (0.23)	2.66 (0.23)
	OA	2.62 (0.23)	2.67 (0.22)	2.67 (0.22)
Overjet (mm)^a^	CBT	2.57 (0.24)	2.57 (0.24)	2.57 (0.24)
	OA	2.95 (0.23)	2.93 (0.23)	2.93 (0.23)
Maximum active right movement (mm)^a^	CBT	9.45 (0.45)	10.05 (0.33)	9.96 (0.37)
	OA	9.35 (0.44)	9.86 (0.33)	9.86 (0.36)
Maximum active left movement (mm)^a^	CBT	10.04 (0.4)	10.57 (0.32)	10.34 (0.38)
	OA	9.74 (0.39)	10.10 (0.31)	10.17 (0.37)
Maximum active protrusive movement (mm)^a^	CBT	9.14 (0.43)	8.88 (0.44)	8.91 (0.42)
	OA	8.66 (0.42)	9.21 (0.43)	8.76 (0.41)
Resiliency of the right TMJ (mm)^a^	CBT	0.56 (0.06)	0.56 (0.06)	0.55 (0.05)
	OA	0.62 (0.05)	0.46 (0.06)	0.36 (0.05)
Resiliency of the left TMJ (mm)^a^	CBT	0.60 (0.07)	0.57 (0.06)	0.59 (0.07)
	OA	0.59 (0.06)	0.43 (0.06)	0.47 (0.06)
Presence of a slide from CO to MI (%)^b^	CBT	64.3	82.1	78.6
	OA	75.9	86.2	82.8
Length of the slide from CO to MI (mm)^b^	CBT	0.53 (0.13)	0.65 (0.11)	0.66 (0.12)
	OA	0.99 (0.12)	0.95 (0.11)	0.82 (0.12)

^a^Repeated measures ANOVA; values are presented as mean and SEM

^b^Friedman test together with Wilcoxon signed rank test; values are presented as mean and SEM

^c^Cochrans Q-test

The statistical analyses of the presence of a slide from CO to MI by means of non-parametric Cochran Q-test, revealed a statistically significant main effect ‘measurement period’ (F (2/57) = 6.93, *p* = 0.031). Contrast analyses revealed a significant increase regarding the presence of a slide from CO to MI from pre-treatment to post-treatment (F (1/57) = 6.40, *p* = 0.01) in both groups (Table 3). No significant interaction between ‘measurement period’ and ‘group’ has been observed. The statistical analyses of the length of a slide from CO to MI by means of non-parametric Friedman test, demonstrated in neither the CBT (Friedman test, χ^2^ = 4.39, *p* = 0.111) group nor in the OA group (Friedman test, χ^2^ = 0.3, *p* = 0.859) statistically significant changes over the three measurement periods. The nonparametric comparison between the two groups merely revealed a statistically significant difference at measurement period pre-treatment (Mann-Whitney *U* test, *p* = 0.023). At baseline, the difference of the mean length of the slide from CO to MI between the OA and the CBT group amounted 0.463 mm, whereas this mean difference decreased at post-treatment and 6 months follow-up (Table 3).

Regarding the resiliency of the right TMJ, a statistically significant main effect ‘measurement period’ (F (1.95/107.14) =7.98, *p* = 0.001, η^2^ = 0.127) was found. Contrast analyses showed a statistically significant reduction of the resiliency of the right TMJ from pre-treatment to post-treatment (F (1/55) = 6.78, *p* = 0.012, η^2^ = 0.110) and from pre-treatment to 6 months follow-up (F (1/55) = 13.61, *p* = 0.001, η^2^ = 0.198) in the OA group, whereas the values recorded in the CBT group remained nearly constant. Furthermore, a statistically significant interaction of the factors ‘measurement period’ and ‘group’ (F (1.95/107.14) = 6.9, *p* = 0.002, η^2^ = 0.111) was calculated. Contrast analyses of the interaction between the aforementioned two factors exhibited a significant change from pre-treatment to post-treatment (F (1/55) = 6.78, *p* = 0.012, η^2^ = 0.110) and from pre-treatment to 6 months follow-up (F (1/55) = 11.52, *p* = 0.001, η^2^ = 0.173) (Table 3). Although both groups showed different courses along the three measurement periods, no significant main effect ‘group’ was detected.

The two-way ANOVA calculated for the variables overbite, overjet, maximum active mouth opening, maximum active left lateral movement of the mandible, maximum protrusive movement of the mandible, and the resiliency of the left TMJ showed neither a statistically significant main effect ‘measurement period’ nor a significant main effect ‘group’. The interaction of the factors ‘measurement period’ and ‘group’ also failed to reveal any statistically significant effect.

## Discussion

The main result of the present study was that regarding the global dimension affective pain perception, SB subjects revealed below average values compared to that of a reference group. The recorded T-values of 37 to 38 for the affective pain perception were located within the first distribution quartile of the reference sample. This means that 80% of the total reference sample (*n* = 1048) report stronger affective pain in the sense of the SES than the investigated SB groups. This outcome refers to the mean values recorded in both treatment groups and wasobserved at all three measurement periods. As regards to the global dimension sensory pain perception, SB subjects showed values at an average and, accordingly, comparable to that of a reference sample. These values were found in both treatment groups and at all three measurement periods. Furthermore, within the three measurement periods statistically significant changes were observed with respect to the functional parameters maximum active right lateral movement and the resiliency of the right TMJ and the subtotal rhythmicity and the global dimension affective pain perception of the SES. Apart from the observed statistically significant effects, it appears noteworthy that the values calculated for all functional/occlusal variables as well as those obtained for the sensory pain perception were clearly located within normative ranges at each measurement period.

To date little is known in terms of the individual pain perception of SB subjects. Moreover, to the authors’ knowledge, this is the first RCT in which possible changes in the individual pain perception of SB subjects treated either with a CBT or a standard dental occlusal appliance were evaluated. For this reason, comparisons of the present findings with those derived from previous investigations could hardly be made. Further arguments that limit comparing the present results with other data are seen in the diversity of the applied pain assessment tools. For instance, *Raigrodski and coworkers* evaluated the effect of the tricyclic antidepressant amitriptyline on pain-intensity level and level of stress in bruxers [47] by using an ordinal scale ranging from 0 to 10, in which “absence of pain” and “worst pain ever” were indicated by 0 and 10, respectively. This randomized, double-blind, crossover experimental study, however, included subjects seeking treatment because of TMD symptoms and affirming at least one of the following questions; do you keep your teeth together? or do you clench or grind your teeth together?. During a 4-weeks administration of either active (25 mg amitriptyline/night) or inactive (placebo 25 mg/night) medication, the mean recordings of subjects daily perception of the level of pain amounted 1.965 (SD 1.533) and 2.825 (SD 1.586) on the 11-point scale. Unfortunately, baseline data on the original perception of the pain level prior to the therapeutic intervention were not reported. Another study investigated the role of parafunctions, emotions and stress in predicting facial pain [12]. This investigation also included subjects with different subgroups of TMDs, who were paged every two hours while awake to fill in a short questionnaire containing rating scales of jaw pain, masticatory muscle tension, time and intensity of tooth contact, mood and stress level. The rating scale used for the estimation of pain levels was again an 11-point (0–10) numerical rating scale. The authors concluded from their results that parafunctional behaviours, especially those that increase muscle tension, and emotional states were good predictors of jaw pain levels in patients with TMDs and healthy control subjects. However, due to the study design it might be assumed that the calculated parafunctions included predominantly awake bruxism and, thus, data cannot easily be compared to that collected in the present investigation which focusses on SB.

In fact, the present study included subjects with SB, but possible awake bruxism was also estimated as a covariable. Approximately two third of the SB subjects also reported awake bruxism. However, due to the fact that the assessment of awake bruxism was solely based on self-reporting by the participants, its value is limited. Nonetheless, similar associations regarding the frequency of a coincidental occurrence of awake bruxism in subjects with SB have been detected by Winocur and coworkers [7]. In this context, attention should be drawn to the process of SB diagnosis used in the present study. As published elsewhere, the current ‘gold standard’ for sleep bruxism diagnosis is represented by the polysomnographic recordings in a sleep laboratory. Undoubtedly, this is due to the good performance of the validity parameters, but they are concomitantly associated with disadvantages which include technical complexity, limited availability [6, 7], and the fact that they are time-consuming and cost-intensive [48]. These disadvantages often result in likewise small sample sizes, which appear from a statistical-methodological point of view as exceedingly problematic. In particular, the polysomnographic recordings are not suitable for clinical trials with a sophisticated study design as implemented in this study; with measurements repeated three times over a period of nine month and a sample size above 50 participants. Therefore, as graded in a recent publication [1, 49], a decision was made to apply the clinical AASM criteria [30] which allow the estimation of ‘probable’ SB.

Another issue that requires discussion is the study design of this interventional approach. Parts of the data concerning the effects of this intervention on SB activity have been published elsewhere [24]. This interventional study compared the effects of a CBT with that of an OA therapy in particular to investigate the possible long-term effects of a CBT. For that reason, both groups required a period of 6 month without any intervention. Moreover, during the period of the intervention additional management approaches, such as the use of an occlusal splint combined with the CBT could have covered the possible effects resulting from the CBT alone. Therefore, the authors found a comparatively manageable treatment period of six month without OA acceptable with respect to the progression of further attrition or possible tooth damage. In search of a perhaps new causal-oriented treatment for sleep bruxism, the authors found the selected study design acceptable as confirmed by the ethical committee of the Medical Faculty. In accordance with the ethical responsibility of scientific investigations, each participant was given the opportunity to try the other therapy at the end of the study period. In the OA group, subjects that required further use of their splint or a new one, received an OA again.

Keeping in mind the below average values obtained for the affective pain perception in the SB subjects of the present study, psychological aspects which are known to have an impact on the intensity of perceived pain [14, 15, 17, 50], need to be taken into consideration. A currently published investigation evaluated the prevalence of psychopathological symptoms in patients who self-reported different forms of bruxism by means of clinical and anamnestic diagnostic criteria [21]. The authors differed between awake bruxers, sleep bruxers, sleep-awake bruxers, and non-bruxers. The analysis of the psychopathological symptoms indicated that patients with sleep–awake bruxism endorsed the highest scores, patients with awake bruxism showed higher scores than patients with SBand non-bruxism in most subscales of the Symptom Check List (SCL-90-R) [51, 52]. Interestingly, in seven of the 9 subscales as well as in the sumscore, viz. the global severity index, sleep bruxers demonstrated values even lower than those of the non-bruxers. Allowing for the indications of an association between psychological factors and the intensity of perceived pain, an interrelation between these comparatively low degrees of psychopathological symptoms in SB subjects and the below average located affective pain perception of SB subjects detected in the present study is supposable. Moreover, this underpins the argumentation of previous authors who point to the opportunity that sensory or affective aspects of pain perception can be modified by techniques that influence variables, such as attentional state, emotional context, empathy, hypnotic suggestions, attitudes and expectations [50].

Concluding from the former, SB might act as a sort of adaptive response to psychological impairment and, consequently, to stress. This assumption is in line with previous authors [22, 40, 53–56], whereas the present study is the first that includes the individual pain perception. Furthermore, within the limitations of this study, it might be assumed that the significantly reduced affective pain perception in SB subjects is the expression of an adaptation mechanism. Additional aspects that support the assumption of SB in a broader sense acting as an adaptation mechanism to psychologic load are the results of the functional/occlusal parameters. Indeed, the statistical analysis from two of the ten recorded variables revealed a significant change during the three measurement periods. These findings were, however, independent of the respectively received therapy, and all recorded values were within normal ranges. Therefore, these changes may be interpreted as the result of physiologic variability and might even be due to the repeated measurement. Although the present findings support the hypothesis that SB might be an adaptive response to psychological impairment and, in particular, to stress, from a methodological point of view they do not allow deriving the conclusion that there is a causal association between below average affective pain perception and SB. In this context, as questioned earlier [40, 46, 57], it becomes of major interest to understand at what point a stomatognathic system or any other peripheral system of the whole body decompensates and turns into a disorder which requires a treatment. Future investigations that take new theories to explain the adaption to pain into consideration [58–60], should focus on the issue evaluating different forms of bruxism and subgroups of TMDs in a controlled longitudinal design in order to gain further scientific knowledge in this field.

## Conclusions

The objectives of the present study were to assess the individual pain perception in SB subjects generally. Furthermore, the effects of a CBT should be compared to an OA therapy on pain perception and, additionally, their possible continuative impact on several functional parameters should be investigated by using a RCT. The main result of the present study was that regarding the global dimension affective pain perception, SB subjects revealed below average values compared to that of a reference group. This outcome refers to the mean values recorded in both treatment groups and was observed at all three measurement periods. Concerning the global dimension sensory pain perception, SB subjects showed values at an average and, accordingly, comparable to that of a reference sample. At all three measurement periods, both treatment groups revealed these values. Furthermore, within the three measurement periods statistically significant changes could be observed with respect to the functional parameters maximum active right lateral movement and the resiliency of the right TMJ and the subtotal rhythmicity and the global dimension affective pain perception of the SES. Apart from the observed statistically significant effects, it appears noteworthy that the values calculated for all functional/occlusal variables as well as those obtained for the sensory pain perception were clearly located within normative ranges at each measurement period.

Within the limitations of this study, it might be assumed that the significantly reduced affective pain perception in SB subjects expresses a kind of adaptive mechanism.

## Abbreviations

AASM: American Academy of Sleep Medicine
ANOVA: Analysis of variance
CBT: Cognitive behavioural therapy
CO: Centric occlusion
GLM: General linear model
ICD: International classification of diseases and related health problems
MI: Maximum intercuspation
OA: Occlusal appliance
RCT: Randomized controlled trial
SB: Sleep bruxism
SEM: Standard error of the mean
SES: Pain perception scale
TMD: Temporomandibular disorder
TMJ: Temporomandibular joint
